# Rapid identification of mycobacteria from positive MGIT broths of primary cultures by MALDI-TOF mass spectrometry

**DOI:** 10.1371/journal.pone.0192291

**Published:** 2018-02-02

**Authors:** Tsi-Shu Huang, Chia-Chien Lee, Hui-Zin Tu, Susan Shin-Jung Lee

**Affiliations:** 1 Division of Microbiology, Dept. of pathology and laboratory medicine, Kaohsiung Veterans General Hospital, Kaohsiung, Taiwan; 2 Division of Infectious Diseases, Department of Internal Medicine, Kaohsiung Veterans General Hospital, Kaohsiung, Taiwan; 3 Faculty of Medicine, School of Medicine, National Yang-Ming University, Taipei, Taiwan; Indian Institute of Technology Delhi, INDIA

## Abstract

**Background:**

Rapid identification of mycobacteria is important for timely treatment and the implementation of public health measures. The MGIT system ensures rapid detection of mycobacteria, but identification is usually delayed by days to weeks due to further subculture on solid medium. Matrix-assisted laser desorption time-of-flight mass spectrometry (MALDI-TOF MS) was demonstrated to effectively identify mycobacteria isolates subcultured from solid or liquid media. Reports of identification directly from MGIT broths of both sterile and non-sterile clinical specimens, omitting the subculture step, were limited and not satisfactory before. Our identification method dramatically shortened delay from detection to identification of mycobacteria.

**Methodology:**

We assessed the performance of the Vitek MS IVD version 3.0 for direct identification of NTM and *M*.*tuberculosis* from primary MGIT cultures, and assessed two sample preparation methods.

**Results:**

Direct identification of NTM from positive MGIT broths, using MALDI-TOF VITEK MS with IVD v.3.0, generated high rates of acceptable results reaching 96.4% (80/83), and up to 100% (83/83) for sample preparations including a 0.1% SDS washing step. The sensitivity of VITEK MS to identify *M*.*tuberculosis* from MGIT tubes was 58/72 (80.6%), when using immunochromatography (ICA) test as gold standard. A characteristic colony clumping, wool-like appearance was observed in 48, and all 58 (100%) were correctly identified as *M*.*tuberculosis* using MALDI-TOF. The detection rate of *M*.*tuberculosis* complex was low (10/24, 41.6%) in the 24 MGIT tubes that was polymicrobial. Our method significantly reduced both the reagent cost and turnaround time.

**Conclusions:**

Based on a simplified protocol, we showed that MALDI-TOF MS can be used for rapid identification of NTM directly from primary MGIT cultures within the routine clinical laboratory workflow. However, we recommend an initial ICA test to screen for *M*.*tuberculosis* complex, due to a low identification rate of *M*. *tuberculosis* in the presence of polymicrobial cultures using MALDI-TOF.

## Introduction

The prevalence of nontuberculous mycobacteria (NTM) infections is increasing, not only in HIV-positive patients, but also in non-HIV patients with underlying lung disease [1–4]. When mycobacteria are recovered from clinical specimens, it is critical to rapidly distinguish *M*. *tuberculosis* complex from NTM, due to major differences with respect to treatment and public health impact [5]. In addition, timely and accurate identification of NTM species facilitate appropriate choice of appropriate antimicrobial therapy [6]. In a 2007 official joint statement by the American Thoracic Society and Infectious Disease Society of America (ATS/IDSA) on the diagnosis, treatment, and prevention of NTM disease, it was specifically recommended that clinically significant NTM isolates should be identified to the species level whenever possible [7]. However, the *Mycobacterium* genus is comprised of more than 150 species [8]. At least 60 NTM species are currently recognized as causative agents of human pathology, with variable severity and prognosis [9, 10]. This recommendation may be challenging for many clinical microbiology laboratories to implement.

The conventional diagnostic tools for mycobacterial infections, microscopy and culture, remains irreplaceable [11, 12]. The Bactec MGIT 960 system, which is fully automated and provides continuous monitoring to identify positive cultures in real time, dramatically reduced the time to diagnosis of mycobacterial infections [13]. However, when a growing bacteria is identified, subculture of the MGIT broth on 7H11 agar plates is still needed to obtain bacterial colonies for further identification. Immunochromatogenic assays (ICAs) provide timely detection of *M*. *tuberculosis* complex immediately after cultures turn positive. It is rapid (readable in 15 min), easy to use, and requires no processing or additional instrumentation [14–19]. But, identification of NTM is still based on a variety of phenotypic tests and enzymatic properties. Such tests are labor-intensive and time-consuming to perform and may take several days to weeks to complete. Moreover, the phenotype method is available for only common species, and may lead to ambiguous or erroneous results [20], that cannot be relied on to guide clinical decisions.

High-performance liquid chromatography (HPLC) analysis of mycolic acid has been used to provide better discrimination between species and a more specific identification [21]. However, this method is not suited to the clinical setting because it is labor-intensive, and instruments required for analysis are not widely available. In addition, it requires a pure culture of isolates on solid medium [21], which delays turnaround time. Molecular methods include PCR-based hybridization and sequencing methods may provide more efficient results, but requires specific technical expertise and is technically demanding. Species-level discrimination by sequencing methods may require comparison of sequences obtained from several genes including 16S rDNA, *rpoB*, and *hsp65*[9, 22]. In addition, similar to HPLC, sequence analysis requires a pure isolate obtained from solid medium and is labor-intensive. PCR-based hybridization directly from liquid culture medium have contributed greatly to shortened turnaround time, however, these also require a high level of expertise and limited species can be identified. INNO-LiPA Mycobacteria (Innogenetics, Ghent, Belgium), targeting the 16S−23S rDNA spacer region, requires expensive equipment [23, 24], but simultaneous detect and identify the genus *Mycobacterium* and 16 different mycobacterial species. GenoType Mycobacterium CM/AS (Hain Lifescience, Nehren, Germany), targeting the 23S rDNA region, allows the detection of 31 species of NTM [25–27].

In recent years, Matrix-Assisted Laser Desorption Ionization Time-of-Flight (MALDI-TOF) mass spectrometry was demonstrated to accurately identify bacteria routinely isolated in a clinical microbiology laboratory [28–30]. The speed, robustness and minimal costs of sample preparation and measurement makes it exceptionally well suited for routine and high-throughput use. It reduces turnaround time and may potentially impact on benefiting patients. Numerous reports have described the performance of the Biotyper (Bruker Daltonics, Germany), Vitek MS RUO (formerly Saramis) and Vitek MS (bioMérieux, France) systems. Both systems use different algorithms for the identification of microbial protein spectra and have been shown to perform similarly for NTM identification [31, 32]. In addition, with regular database expansion, the identification efficiency can be further enhanced. The latest released MBT Mycobacteria RUO Library version 5.0 on the Bruker Daltonic platform covers 164 of the currently known 180 mycobacteria species. However, the database is not FDA approved. VITEK MS contains a FDA-approved database IVD v.3.0 that includes 45 *Mycobacterium* species. In both databases, species belonging *Mycobacterium tuberculosis* complex (MTBC) allows only complex-level identification. For routine use, user friendliness is an important consideration in a clinical laboratory. The availability of disposable targets and ready-to-use matrix solution of the Vitek MS system reduce pre-analytical steps and possible errors. In addition, Vitek MS is easier to integrate into the workflow, using a common middleware (Myla^™^, bioMérieux) with other routine devices in our lab (Vitek 2, bioMérieux).

Previous studies showed that reliable identification to species can be done using mycobacteria obtained from subculture on solid-phase medium, such as the Lowenstein-Jensen or Middlebrook 7H10 or 7H11 medium [31–38]. Identification rates using a Biotyper system was 77% [39] and detection rate increased from 67% to 94% by using the VITEK MS system for isolates grown on liquid media MGIT, using Saramis v4.12 and IVD v3.0 [40]. However, these studies all used mycobacterial isolates subcultured from either solid or liquid medium. In contrast, our study used mycobacteria isolates from MGIT broths, omitting the subculture step in identification. There are very limited number of studies reporting identification using isolates from MGIT medium without subculture, and showed that identification rates of NTM were poor when using a liquid medium [39–41]. Currently, there is only one published report using MALDI-TOF MS to identify NTM from 53 newly positive, liquid cultures of respiratory samples, and demonstrated a low rate of correct identification rate of 22% [42]. Identification of mycobacteria directly from MGIT broths can dramatically shorten the delay between detection by culture positivity to definitive identification of the mycobacteria. We therefore aimed to assess the performance of MALDI-TOF MS analysis for direct identification of *Mycobacterium* spp. from positive MGIT broths without subculture, compared to the routine protocol (with subculture), under real-world, routine laboratory settings.

## Materials and methods

The study protocol was approved by the Institutional Review Board of Kaohsiung Veterans General Hospital, (No. 17-CT9-04).

### Samples

We prospectively analyzed MALDI-TOF identification of mycobacteria directly from culture positive MGIT broths compared with the routine protocol, from Dec. 2016 to May 2017. All samples submitted for mycobacterial cultures were inoculated into the MGIT broth and cultured with MGIT Bactec MGIT 960 instrument (Becton Dickenson Cockeysville, Maryland, US) as well as Lowenstein-Jensen medium and incubated at 35°C.

Tubes flagged positive by the MGIT 960 instrument were removed from the instrument, subjected to the acid fast stain to evaluate the growth appearance as purity check and ICA (SD Bioline Ag MPT64 Rapid assay) test for rapid detection of *M*. *tuberculosis* complex. Positive MGIT broths that are negative for *M*.*tuberculosis* antigen using ICA were kept in room temperature for further identification to the NTM species at a monthly interval if considered clinically relevant [43]. Identification of MTB and NTM to species level by VITEK MS from the culture positive MGIT broth was compared to the results obtained by ICA test and Microchip array (Dr Chip Biotech Inc., Miao-Li, Taiwan), respectively, in the 6-months study period. All three methods were performed using the same liquid culture.

### Sample preparation for MALDI-TOF Vitek MS

For identification of NTM, 1 ml of culture broth was used for both sample preparation methods (the direct method and the SDS method). To ensure sufficient amount of bacteria in the samples, the test was repeated using up to 3mL of the same MGIT culture broths, if the first try using 1mL failed. For identification of *M*. *tuberculosis*, 3 ml was used for both methods.

The positive MGIT culture broths were vortexed for 5–10 seconds and 1 ml broth was transferred to a 1.5 ml Eppendorf tube. The Eppendorf tube was centrifuged at 14,000 rpm for 3 minutes, then the supernatant was completely removed. The pellet was either (a) subjected to ethanol inactivation without any washing step (the direct method), or (b) washed with 500 μL 0.1% Sodium Dodecyl Sulfate, centrifuged again at 14,000 rpm for 3 min before ethanol inactivation (the SDS method). The bacterial pellet was re-suspended with 500 μL of 70% ethanol and transferred to another 1.5 ml Eppendorf tube with 200 μL 425–600 um glass beads (SIGMA). After vortexing for 15 min and keeping in room temperature for 10 more minutes, the bacterial suspension was transferred to another 1.5 mL Eppendorf tube, centrifuged for 3 min to completely remove the supernatant. The pellet was mixed with 10 μL 70% formic acid by vortex for 3–5 seconds, then 10 μL 100% acetonitrile was added and mixed again by vortex for 3–5 seconds. The suspension was centrifuged at 14,000 rpm for 2 minutes and moved out of biosafety level III facility. One μL of the supernatant was spotted onto a MALDI-TOF target plate and air dried.

### MALDI-TOF MS

Each deposit on the target plate was overlaid with 1 μL of matrix (VITEK MS CHCA) and air dried. The slide was run in the MALDI-TOF instrument (bioMérieux VITEK MS) to obtain the identification. All organisms were placed onto only one well of a Vitek MS slide. Target plates were calibrated and quality controlled both before and after data acquisition by using Escherichia coli ATCC 8739. After the acquisition of spectra, data were transferred from the Vitek MS acquisition station to the Vitek MS analysis server, and identification results were displayed using Myla v2.4 middleware. Each operator participating in the study was required to analyze a proficiency panel successfully prior to beginning to test isolates for this investigation.

### Microchip array MTB assay

Microchip array MTB assay was performed according to the manufacturer's directions. Briefly, 0.5 ml of MGIT broth was washed and sonicated at 50°C for 30 minutes. The vial was heated in boiling water for 20 minutes and then chilled on ice for 5 more minutes. After removal of cell debris, 5 ul aliquots of the extracted DNA were added to 20 μL of master mix and placed in a thermal cycler to perform the following program: 95°C for 4 min (1 cycle); 95 °C for 20 s, 55 °C for 20 s, and 72 °C for 40 s (15 cycles) and 95 °C for 20 s, 60 °C for 20 s, and 72 °C for 40 s (25 cycles); extension at 72 °C for 5 min. Mix 90 μL DR. HybTM Buffer and 10 μL amplicons in a new 1.5 mL Eppendorf vial. The vial was heated in boiling water for 5 minutes and then chilled on ice for 5 minutes immediately. The hybridization mixture was transferred into each well and covered with the plastic membrane. The plate was incubated at 55°Cwith vibration for 1 hour in DR. Hyb^™^ oven (DR. Chip). The plastic membrane was removed, then placed on DR. Fluidic Station and execute “TB-SC” program under DR. Fluido software. The patterns were read and analyzed with DR. AiM Reader (DR. Chip).

### Discordant results

Samples with discordant results between MS system and Microchip MTBC assay were further identified by polymerase chain reaction-restriction enzyme analysis (PRA). BstEII and HaeIII enzyme digestion of the amplification product was performed as described previously [44] and compared with PRA profiles published [10, 45–47].

### Cost estimates

We evaluated the cost per isolate for the MALDI-TOF mass spectrometry identification by adding the costs of Matrix reagents, target plates, and positive controls. All cost estimates are in U.S. dollars and reflect the actual costs incurred in our laboratory and not charges to the patient.

## Results

**Fig 1:**
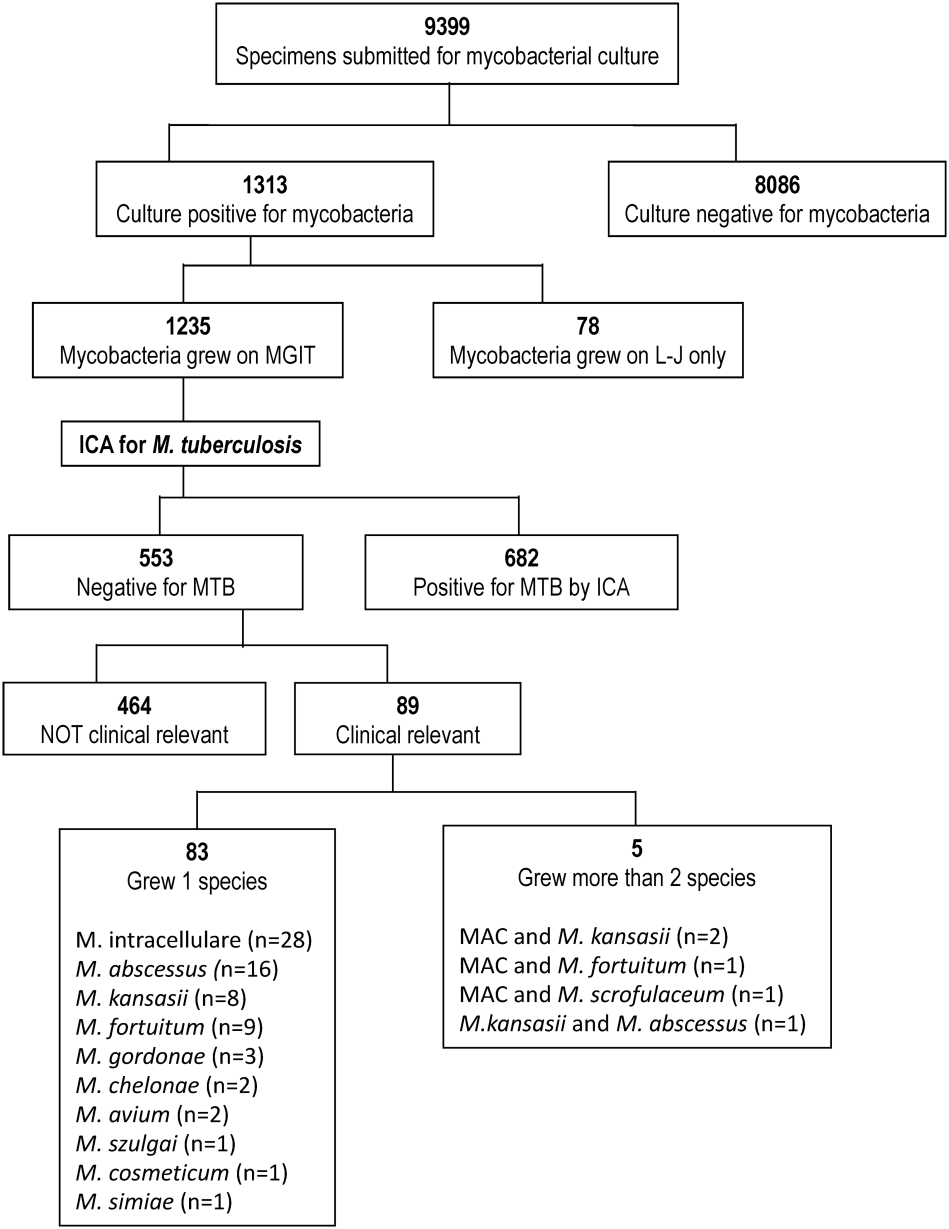
The flow of selection of specimens. Diagram showing the flow of selection of specimens included in this analysis, including the methods used and the mycobacterial species isolated. (MAC, M. avium-intracellulare complex).

A total of 9399 clinical specimens were submitted to our laboratory from Dec 2016 to May 2017 and cultivated in both liquid (MGIT and BACTEC 460) and solid Lowenstein-Jensen) media. Cultures were positive for mycobacteria in 1313 specimens, of which 1235 grew on MGIT broths. Among them, 682 (55.2%) were detected positive for *M*.*tuberculosis* by the ICA test. The remaining 553 MGIT broths recovered nontuberculous mycobacteria. Direct identification of culture-positive MGIT broths, detected 72 MGIT broths positive for *M*.*tuberculosis* and 89 recovered NTM that was considered clinically relevant and subjected to further identification. Among the 89 broths that recovered NTM, 5 was polymicrobial and 83 monomicrobial. NTM isolates identified included *M*. *avium-intracelluare* complex (MAC; n = 30), *M*. *abscessus* (n = 16), *M*. *kansasii* (n = 8), *M*. *fortuitum* (n = 9), *M*. *gordonae* (n = 3), *M*. *chelonae* (n = 2), and one each of *M*. *szulgai*, *M*. *cometicum*, *M*. *mageritense and M*. *simiae*. (Fig 1). The NTMs were recovered from 70 respiratory specimens (including 59 sputum, 8 gastric lavage, lung tumor 1, bronchial washing 1 and BAL fluid 1) and 13 extrapulmonary specimens (including 4 pleural fluid, 5 pus/wound, blood 1, synovial fluid 1, urine 1 and tissue 1).

Correct identification of NTM was obtained in 80 (96.4%) MGIT tubes by using the Microchip MTB assay. *M*. *cometicum* was misidentified as *M*. *neoneunm* by the Microchip MTB assay. Additionally, identification failed in two isolates, *M*. *intracellulare* and *M*. *mageritense*.

Analysis of positive MGIT broths using MALDI-TOF VITEK MS with IVD v.3.0 generated identification rates of 86.7% (72/83) and 89.2% (74/83) using the direct and SDS methods, respectively. (Table 1). MGIT broths that had MALDI-TOF results showing “bad spectrum” or “no identification”, had a smaller pellet size after centrifugation than those with correct identification.

**Table 1:** Rates of correct identification of different mycobacterial species using the conventional Microchip MTB method compared to direct identification from positive MGIT broths using the MALDI-TOF MS Vitek MS system with a IVD database (v3.0) prepared without any washing step (Direct) and washed once with 0.1% SDS (SDS) protocol (N = 83).

Species	No. of samples		No. of samples identified:			
			First		Repeat	
		Microchip MTB n (%)	Direct n (%)	SDS n (%)	Direct n (%)	SDS n (%)
MAC*	40	39 (97.5)	30 (75.0)	32 (80.0)	37 (92.5)	40 (100.0)
*M*. *abscessus*	16	16(100.0)	16 (100.0)	16 (100.0)	16 (100.0)	16 (100.0)
*M*. *kansasii*	9	9 (100.0)	8 (88.9)	8 (88.9)	9 (100.0)	9 (100.0)
*M*. *fortuitum*	9	9 (100.0)	9 (100.0)	9(100.0)	9 (100.0)	9 (100.0)
*M*. *chelonae*	2	2 (100.0)	2 (100.0)	2 (100.0)	2 (100.0)	2 (100.0)
*M*. *szulgai*	1	1 (100.0)	1 (100.0)	1 (100.0)	1 (100.0)	1 (100.0)
*M*. *gordonae*	3	3 (100.0)	3 (100.0)	3 (100.0)	3 (100.0)	3 (100.0)
*M*. *cosmeticum*^$^	1	0	1 (100.0)	1 (100.0)	1 (100.0)	1 (100.0)
*M*. *simiae*	1	1 (100.0)	1 (100.0)	1 (100.0)	1 (100.0)	1 (100.0)
*M*. *mageritense*	1	0	1 (100.0)	1 (100.0)	1 (100.0)	1 (100.0)
**Total**	**83**	**80 (97.6)**	**72 (86.7)**	**74 (89.2)**	**80 (96.4)**	81 (100*)

*MAC: *M*. *avium-intracellulare* complex

^*$*^Identified as *M*. *neoneunm* by Microchip MTB; *M*. *cosmeticum* by MS and PRA.

In the routine workflow in our laboratory, positive MGIT tubes that were negative for *M*.*tuberculosis* using the ICA test, were kept in room temperature. NTM identification was performed monthly upon request or if the clinical criteria for NTM disease is met. As a result, MALDI-TOF MS was performed using cultures of different ages. Table 2 shows the rate of correct identification and the no of samples with different culture ages when identification was done, defined as the number of days between first detection by a positive MGIT culture to when identification testing was done. Although the age of the culture may influence the MALDI-TOF MS spectra, this delay did not affect the species-specific profiles. All the rapidly growing mycobacteria (n = 29) were correctly identified, with no association with the duration of culture positivity. However, in slow growing mycobacteria, a higher identification rate was associated with an increase in the number of days of culture positivity. Identification rate was increased to 96.4% (80/83) and 100% (83/83) for direct and SDS methods, respectively, when a larger sample size was used (up to 3mL of the same MGIT broth), producing a bigger pellet size of more than 2 mm along the side of Eppendorf tube. Notably, Microchip MTBC assay was unable to discriminate among the MAC complex. In contrast, MALDI-TOF VITEK MS was able to identify the 29 MAC strains to be 2 *M*. *avium* and 38 *M*. *intracelluare*.

**Table 2:** The distribution of the age of the culture at identification testing of different mycobacterial species, defined as the number of days between detection by a positive MGIT to when identification of was performed and the rates of correct identification of different mycobacterial species.

Species	Total No.	No. of samples tested at different culture ages (days between a positive MGIT to identification testing)			
		0–6	7–13	14–20	>21
Rapid growing mycobacteria	29	5	8	9	6
* M*. *abscessus*	16	2	5	6	3
* M*. *chelonae*	2		1		1
* M*. *fortuitum*	9	2	2	3	2
* M*. *cosmeticum*	1				
* M*. *mageritense*	1	1			
*** *Subtotal**	**29**	**5**	**8**	**9**	**6**
*** *Correct identification rate**		**100%**	**100%**	**100%**	**100%**
Slowly growing mycobacteria	54	9 (3)	13 (2)	13 (2)	19
* M*. *avium-intracellulare* complex	40	7 (2)	9 (1)	11 (2)	13
* M*. *kansasii*	9	1	4 (1)	1	3
* M*. *szulgai*	1				1
* M*. *simiae*	1				1
* M*. *gordonae*	3	1 (1)		1	1
*** *Subtotal**	**54**	**9 (3)**	**13 (2)**	**13 (2)**	**19**
*** *Correct identification rate**		**66.7%**	**84.6%**	**84.6%**	**100%**
**Total**	**83**	**14 (3)**	**21 (2)**	**23 (2)**	**25**
**Correct identification rate**		**78.6%**	**90.5%**	**91.3%**	**100.0%**

For the 72 MTB-positive MGIT tubes, 3 mL of MGIT broths were taken for MS analysis. In the 48 MGIT tubes with typical “cotton wool-like” macroscopic morphology that suggested pure cultures [48], all were correctly identified. (Table 3) The other 24 MGIT tubes with other macroscopic morphology due to polymicrobial cultures, only 10 (41.7%) were detected as *M*.*tuberculosis*. The other 14 broths were identified as *M*. *genavense* (n = 5, 6.9%), *M*. *intracellulare* (n = 3, 4.2%), *Stenotrophomonas maltophilia* (n = 1, 1.4%), and the other 5 (6.9%) was unidentified.

**Table 3:** The macroscopic features of different mycobacterial species in the 72 MGIT tubes which was positive for *M*. *tuberculosis* complex by using immunochromatogenic assay, and later identified by MALDI-TOF MS Vitek MS system with a IVD database (v3.0).

Mycobacteria identified by MALDI-TOF MS system	Macroscopic morphology in MGIT tubes		
	Total n (%)	Cotton wool	Other morphology
Total	72	48	24
*M*. *tuberculosis* complex	58 (80.6%)	48	10
*M*. *genavense*	5 (6.9%)	0	5
*M*. *intracellulare*	3 (6.9%)	0	3
*S*. *maltophilia*	1 (6.9%)	0	1
No identification	5 (6.9%)	0	5

**Fig 2:**
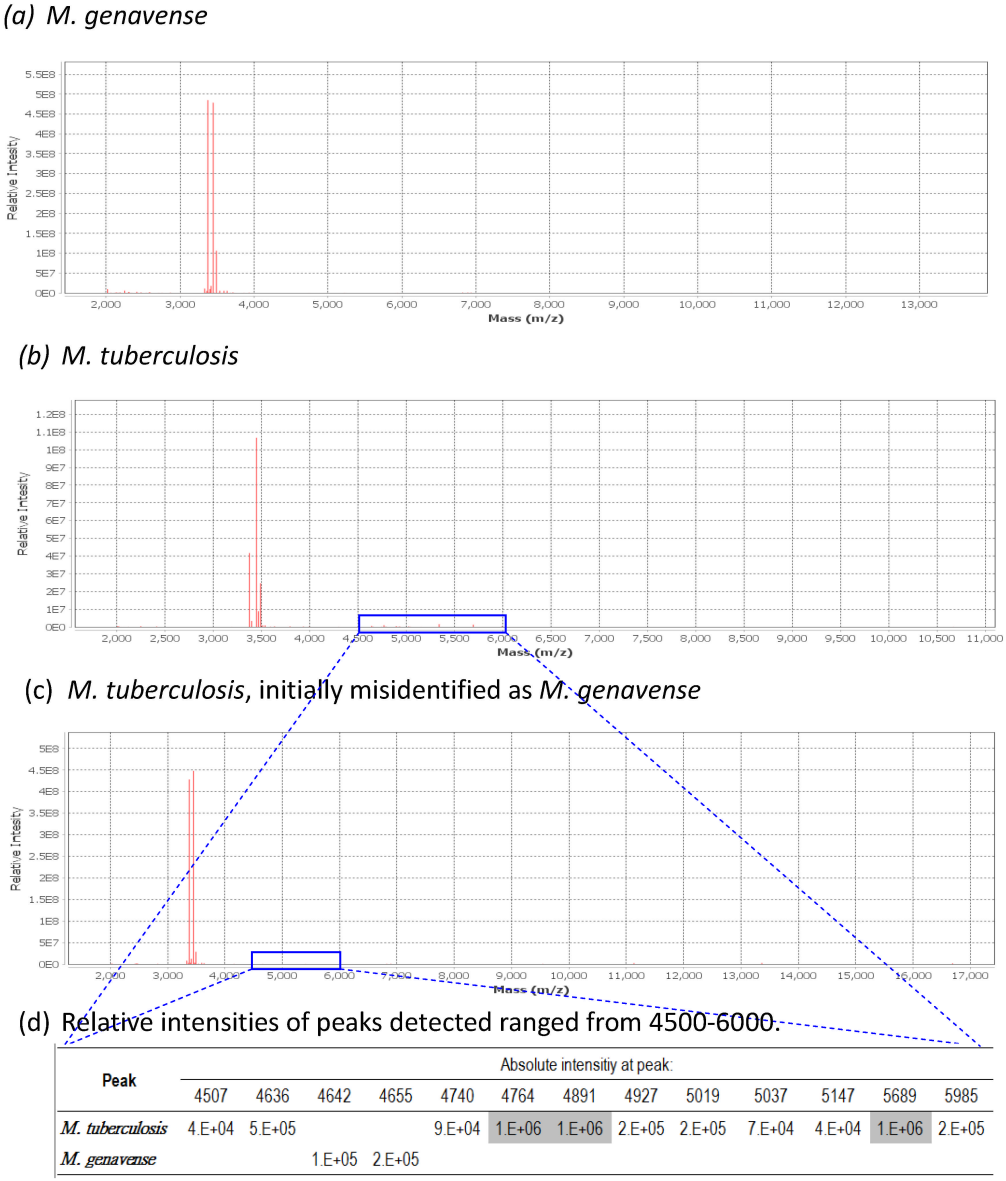
MALDI-TOF MS mass spectrum obtained from *M*. *genavense* and *M*. *tuberculosis*. The mass spectra in (a) and (b, c) were obtained from *M*. *genavense* and *M*. *tuberculosis*.. Panel (b) and (c) were repeated using the initial positive MGIT tube which was misidentified as *M*. *genavense* (c). The spectra in (b) was generated using the same extraction method, with increased number of bacterial cells, showing minor peaks visible in the spectrum ranged from 4500–6000 as shown in gray shading (c).

Six MGIT broths that initially identified as *M*. *genavense* were confirmed to be *M*. *intracellulare* (1) and MTB (5). Fig 2 shows that *M*. *genavense* has very few discriminating peaks, and resembled the peak profiles of some *M*.*tuberculosis* and *M*. *intracellulare*.

### Cost and turnaround time

The reagent cost-per-bottle for direct identification from MGIT broth by VITEK MS compared to by microchip assay was reduced from US$12.4 to US $3.6. The turnaround time was reduced approximately from 4 hours to 60–70 minutes.

## Discussion

Our study demonstrated that direct identification of NTM from a positive MGIT culture broth without subculture using VITEK MS was comparable to the Microchip MTB assay. VITEK MS correctly identified 96.4% of 83 positive MGIT broths with single isolates, which increased to 100% after treatment with 0.1% SDS; and Microchip MTB assay correctly identified 97.5%.

One recent report which used MALDI-TOF MS to identify NTM from 53 newly positive liquid cultures of respiratory samples that were all monomicrobial, demonstrated a low rate of correct identification rate of 22% [42]. In that report, extraction started within 24 to 48 hours after the MGIT tube was flagged positive and only 1.2 mL of culture fluid was used for direct MALDI-TOF MS analysis. Only 7 (13.2%) were rapidly growing mycobacteria. In our study, rapidly growing mycobacteria were all identified successfully in the first week. Slowly growing mycobacteria had correct identification rate of 66.7% (6/9), and the rate increased with the duration that the MGIT broths were kept in room temperature. This implied that failure to identify mycobacteria using MALDI-TOF may be due to insufficient amount of bacteria in the sample. After increasing the volume of the culture fluid to 3 mL, high rates of identification rates were obtained. This was further strengthened by the protocol released recently by Biomerieux which suggests the use 3 mL of culture fluids for analysis.

In addition, we also demonstrated that the identification rate can be further increased to 100% by washing the bacteria pellet using 0.1% SDS. Therefore, we suggest that the optimal sample preparation method is to use 3 mL of culture fluid and sample treatment with 0.1% SDS.

The overall identification rate of *M*. *tuberculosis* from MGIT tubes by VITEK MS was lower (80.6%) than ICA. In pure cultures of *M*. *tuberculosis*, where typical cotton wool-like macroscopic appearance was observed in MGIT tubes, correct identification reached 100%. A low rate of identification (41.7%) was achieved when MGIT tubes were polymicrobial, where isolation of *M*. *tuberculosis* was mixed with NTM or other bacteria on subculture. A known limitation of MALDI-TOF MS is its inability to identify individual components in a polymicrobial culture. In countries where positive NTM cultures is frequent, there is a high possibility of isolating NTM in addition to *M*.*tuberculosis* complex in TB patients, which may lead to incorrect results with masking of TB.

The reported incidence of positive NTM cultures was between 14.1–20.3 per 100,000 persons in year 2000–2003 in urban areas, such as Taipei[4], Ontario[49], and New York city[2]. The recovery of more than one mycobacteria from MGIT broths were not uncommon. Therefore, we suggest that MALDI-TOF analysis be used only for NTM identification after exclusion of *M*. *tuberculosis* by a negative ICA.

*M*. *genavense* is a newly described pathogen with high levels of relatedness with *M*. *triplex* [50]. It causes disseminated infections in patients with AIDS. The clinical features mimicked those of disseminated *M*. *avium* complex infection, with invasion of liver, spleen and lymph nodes with acid-fast bacilli (AFB).[51] However, in our study, the initial identification of 6 strains of *M*. *genavense* was reconfirmed to be *M*. *intracellulare* (5) and *M*. *tuberculosis* complex (1). The misidentification may due to a low number of bacterial cells producing few signature molecules, resulting in peak profiles that mimics some *M*.*tuberculosis* and *M*. *intracellulare*, since *M*. *genavense* has very few discriminating peaks. We therefore suggest that identification of *M*. *genavense* should always be reconfirmed.

The extremely high speed and low marginal cost of MS may improve laboratory efficiency if used directly on new positive liquid cultures. Identification by MALDI-TOF VITEK MS can be completed in approximately 1 hour after the MGIT broths were detected to be positive. Although it is not possible to perform sample preparation for MS each time mycobacteria is detected positive by the MGIT960 system without full automation of the procedure, it is feasible to perform the procedure once per day since the protocol can fit easily into the clinical laboratory workflow.

NTM is ubiquitous in the natural and healthcare environment, and the clinical significance of isolating NTM in clinical specimens is difficult to determine. Rapid and accurate identification to the species-level may aid in guiding management decisions based on the pathogenic potential of the isolated species. Direct identification of NTM from positive MGIT culture broths can significantly shorten the turnaround time.

The efficiency of mass spectrometry to identify mycobacteria grown on solid or liquid media was recently confirmed as a promising technique. VITEK MS v3.0 platform is an IVD system validated by manufacturer and provides many benefits. First, mycobacteria inactivated method recommended by the manufacturer only utilized simple materials, glass beads and 70% ethanol, to achieve safe, fast and effective inactivated results[52]. VITEK MS contains IVD-CE marked database for 49 species of mycobacteria, including 4 species in *M*. *tuberculosis* complex (*Mycobacterium tuberculosis*, *Mycobacterium africanum*, *Mycobacterium bovis*, and *Mycobacterium canettii*) and appeared relatively unaffected by the extraction method [33]. In this study, *M*. *cometicum* and *M*. *mageritense* were not included in the Microchip MTB database.

Finally, we performed a cost analysis to determine whether the VITEK MS is economically competitive in the diagnostic laboratory. The reagent cost of MALDI-TOF was lower compared to the microchip assay, with a reduction from US$12.4 to US $3.6. There was a shortened turnover time from 4 hours using the microchip assay, to an average hands-on-time of 60–70 min per isolate for identification using MALDI-TOF [53, 54]. Although the use of MALDI-TOF requires more expensive equipment and higher maintenance costs, the same system can be used for bacterial and yeast identification, which can reduce overall cost. In addition, the cost for identification instruments, supplementary biochemical tests and quality control testing for bacteria, yeast and mycobacteria can be reduced. It is important to take into consideration that the earlier identification of mycobacteria infections can result in other potential cost-savings, such as shorter hospital stays or better patient outcomes. Furthermore, VITEK MS runs user-friendly platform such as ready-to-use reagents, simplicity of operation [55]. These benefits may improve the workflow and turnaround time of mycobacterial identification and provide comprehensive operational requirement of clinical microbiology laboratory.

The limitation of this study was that only patients that were considered potentially clinically relevant were included into the study for further identification of the positive MGIT broths. Mycobacteria species that were considered to be environmental mycobacteria with low pathogenic potential were not studied. However, this is the group of patients that are clinically important and requires identification of NTM to the species level.

In conclusion, we demonstrated that the use of VITEK MS with IVD v3.0 is a reliable method for identifying NTM to the species level directly from culture positive MGIT broths without requiring subculture, which significantly shortened the turnaround time. We proposed an improved method for extraction of mycobacterial proteins using a bead-based disruption and the addition of 0.1% SDS washing step which achieved high rates of correct identification. However, we recommend that the detection of *M*. *tuberculosis* should be done initially by using ICA because it is easy to perform, can be done within the BSL-3 facility, and most importantly, its performance is not influenced by the presence of multiple bacteria in the specimen. We also showed that the most important factor for correct identification by MALDI-TOF is the amount of bacteria and not the freshness of the culture fluid. Our results provides promise for application and incorporation of MALDI-TOF MS into routine use in the clinical setting for rapid identification of NTM directly from culture positive liquid MGIT broths.
